# Severe Combined Immunodeficiency: Knowledge and Information Needs Among Healthcare Providers

**DOI:** 10.3389/fped.2022.804709

**Published:** 2022-02-21

**Authors:** Oksana Kutsa, Angela Gwaltney, Alissa Creamer, Melissa Raspa

**Affiliations:** ^1^GenOmics, Bioinformatics, and Translational Research Center, RTI International, Research Triangle Park, NC, United States; ^2^Immune Deficiency Foundation, Towson, MD, United States

**Keywords:** severe combined immunodeficiency, information needs, healthcare providers, newborn screening, knowledge gaps, rare genetic conditions

## Abstract

**Background:**

Severe combined immunodeficiency (SCID) is a group of life-threatening genetic disorders responsible for severe dysfunctions of the immune system. Despite the expansion of newborn screening in the U.S., there are gaps in healthcare providers' knowledge of SCID.

**Methods:**

We recruited 277 U.S. healthcare providers for an online survey. The survey assessed providers' experience with SCID patients, knowledge about SCID, and needs and preferred formats for SCID-related informational resources. We examined differences between providers who have seen 2 or more patients with SCID (SCID provider group) and those who have seen 0–1 SCID patients (non-SCID provider group).

**Results:**

Overall, 210 (75.8%) providers were included in the non-SCID provider group, and 121 (57.6%) of these providers were pediatricians. Compared to the SCID provider group, non-SCID provider group reported lower mean rating of SCID knowledge (x̄ = 4.8 vs. x̄ = 8.6, *p* < 0.0001) and higher informational needs. The largest informational needs identified by the non-SCID provider group were “understanding specific type of SCID” and “understanding what to expect across the lifespan.” In the SCID provider group, the highest rated informational need was “family support referrals.” Participants in the non-SCID provider group identified scientific publications and websites as preferred formats, with some variation between medical specialties.

**Conclusion:**

Based on their experience with treating SCID patients, providers have varying levels of SCID knowledge and different informational needs. For providers who have encountered few SCID patients, informational needs start early, usually immediately after receiving a positive newborn screening result. These findings provide useful direction for the development and preferred outlets for receiving SCID-related information, with some variations between different types of providers. Results from this study will serve as a guide for creating relevant and accessible SCID resources for providers who can utilize them to improve care for SCID patients.

## Introduction

Severe combined immunodeficiency (SCID) is a group of genetic disorders characterized by defects in cellular and humoral immunity (1, 2). Patients with SCID have a deficiency or absence of T-cells and are highly susceptible to opportunistic and recurrent infections. Without immune reconstituting treatments, patients diagnosed with SCID do not survive infancy (3). Today, early identification and treatment are effective at reducing SCID-related morbidity and mortality (1, 2, 4). SCID is considered a rare disease, with an estimated prevalence of 1 in 58,000 births (1, 5).

Before the implementation of nationwide newborn screening for SCID in 2018, clinical diagnosis often was delayed due to absence of family history and lack of distinguishing symptomology at birth. Despite the expansion of newborn screening, there is a gap in knowledge among healthcare providers about the management of rare genetic conditions like SCID. A survey of pediatric residents in the US reported that 56% were not aware of appropriate follow-up for abnormal newborn screening results (6). Over 40% of pediatricians reported they were unprepared to talk about newborn screening results with families (7). In a qualitative study of cystic fibrosis screening, primary care providers disclosed that delivering screening results to parents was challenging and required determining the content of the initial conversation and practicing addressing parents' questions (8). More globally, healthcare providers in Spain identified that major challenges in the care of patients with rare genetic diseases are scarce diagnostic guidelines, inability to make a definitive diagnosis, and uncertainty about how to refer patients to specialty follow-up (9). Similar gaps in provider knowledge have been reported in Poland, Australia, Italy, and the Netherlands (10–13).

To date, there are no studies evaluating U.S. healthcare providers' knowledge about management of patients with SCID. The purpose of this study was to (1) assess the healthcare providers' knowledge about SCID and comfort in meeting the needs of patients with SCID, and (2) understand informational needs and preferred formats for SCID-related educational resources among providers. By analyzing SCID-related knowledge and informational needs among healthcare providers, the results of this study will ultimately help to provide informed care and appropriate resources to patients and families with SCID.

## Materials and Methods

### Participants

Healthcare providers in the U.S. were recruited to take part in an anonymous, online survey. To target healthcare providers who were more likely to have experience treating patients with SCID, the Immune Deficiency Foundation (IDF) and SCID Angels for Life distributed the survey announcement to providers on their mailing lists. In addition, an online panel company (M3) was contracted to help recruit a broader group of general practitioners (e.g., pediatricians, family medicine providers, nurse practitioners) who were less likely to see patients with SCID.

Study participants were stratified into two groups based on the self-reported number of SCID patients seen: those participants who had seen 0–1 patients with SCID (median = 0, range = 0–1) were grouped into the non-SCID provider group, and those participants who had seen 2 or more patients with SCID (median = 10, range = 2–99) were included in the SCID provider group. There were only two providers who had seen one patient, one of which who had seen a patient with SCID in the last year and the other who had seen a patient with SCID more than 5 years ago. This stratification allowed for examination of information needs and preferences for formats of educational materials by those more and less familiar with treating a patient with SCID. Out of 359 respondents, 277 providers met the inclusion criteria and completed the survey and thus were included in the analyses (non-SCID provider: *n* = 210, SCID provider: *n* = 67).

### Instruments

The survey contained three sections. In the first, participants were asked to provide demographic information, including sex, race, ethnicity, age, medical specialty, years of experience, and practice setting (e.g., academic hospital, private practice). Items in the second section asked about the provider's experience with SCID patients, including whether they had treated a patient with SCID, how many patients treated in total, and how recently they provided care to a patient with SCID. Additional questions asked about knowledge of SCID and comfort in meeting the needs of patient with SCID, both of which were rated on a 10-point Likert-type scale, with higher ratings indicating more knowledge or comfort. The final section of the survey asked about resources needs. The first few items focused on whether the providers had sought additional information about SCID and if so where (e.g., other providers, peer-reviewed literature, professional organizations). Next, providers were shown a matrix of information needs that they may have or could need related to SCID and asked to rate whether each would be “not a need,” “a small need,” or “a large need.” Finally, providers were asked to rate their preference for receiving information about SCID through a variety of methods, such as reading a website or a professional publication (7-point Likert-type scale, with higher ratings indicating more interest). Data collection was open for 6 weeks.

### Data Analysis

Descriptive analysis was conducted using frequencies, means, and standard deviations. Differences between measurement values of the two groups were calculated using the independent sample *t*-test according to the distribution, and the χ^2^ test for categorical variables or Fisher's Exact test where cell sizes are small. ANOVA and Tukey's studentized range tests were used to determine mean differences between informational formats. A *p* < 0.05 was considered statistically significant. SAS v.7.1 was used in the analysis of the survey data (Cary, NC; 2017).

## Results

### Demographics

Of the 277 study participants, about a quarter (*n* = 67, 24.2%) had treated two or more patients with SCID (Table 1). Respondents in the SCID provider group were, on average, 7 years older (*p* < 0.0001) and had 5 more years of experience (*p* < 0.01) than the respondents in the non-SICD provider group. More than half of the participants in the non-SCID provider group were pediatricians (*n* = 121, 57.6%), and most of the SCID-provider group consisted of allergists/immunologists (*n* = 42, 62.7%). Most of the participants in the non-SCID provider group worked in private practice (*n* = 92, 43.8%), whereas over three-quarters of the participants in the SCID-provider group (*n* = 51, 76.1%) worked in an academic hospital setting. There were no differences between the two provider groups on sex, race, or ethnicity.

**Table 1:** Demographics.

		**All**		**Non-SCID provider group**		**SCID provider group**		***p*** -value*
		***N*** **= 277**		***N*** **= 210**	**(75.8%)**	***N*** **= 67**	**(24.2%)**	
		***N***	**(%)**	***N***	**(%)**	***N***	**(%)**	
Sex								NS
	Female	152	(54.9)	111	(52.9)	41	(61.2)	
	Male	124	(44.8)	98	(46.7)	26	(38.8)	
Age								
	Mean (SD)	45.6	(11.5)	43.9	(10.4)	51.3	(13.3)	<0.0001
Race								NS
	American Indian or Alaska	1	(0.4)	1	(0.5)	·		
	Asian	49	(17.7)	35	(16.7)	14	(20.9)	
	Black or African American	7	(2.5)	7	(3.3)	·		
	White	196	(70.8)	145	(69.1)	51	(76.1)	
	More than one race	10	(3.6)	9	(4.3)	1	(1.5)	
	Prefer not to say	13	(4.7)	13	(6.2)	·		
Ethnicity								NS
	Hispanic or Latino	11	(4.0)	8	(3.8)	3	(4.5)	
	Not Hispanic or Latino	250	(90.3)	188	(89.5)	62	(92.5)	
	Prefer not to say	14	(5.1)	14	(6.7)	·		
Primary role or specialty								<0.0001
	Allergist/immunologist	45	(16.3)	3	(1.4)	42	(62.7)	
	Bone marrow transplant physician	6	(2.2)	·		6	(9.0)	
	Family medicine physician	31	(11.2)	31	(14.8)	·		
	Genetic counselor	22	(7.9)	21	(10.0)	1	(1.5)	
	Infusion specialist	2	(0.7)			2	(3.0)	
	NBS Follow-up	3	(1.1)	3	(1.4)			
	Nurse practitioner	20	(7.2)	16	(7.6)	4	(6.0)	
	Oncologist/hematologist	1	(0.4)	·		1	(1.5)	
	Pediatrician	123	(44.4)	121	(57.6)	2	(3.0)	
	Physician assistant	16	(5.8)	15	(7.1)	1	(1.5)	
	Rheumatologist	1	(0.4)	·		1	(1.5)	
	Other	7	(2.5)			7	(10.5)	
Years of experience in current role								
	Mean (SD)	14.1	(9.8)	12.9	(8.2)	17.9	(13.0)	<0.01
Practice Setting								<0.0001
	Academic hospital	107	(38.6)	56	(26.7)	51	(76.1)	
	Community clinic	30	(10.8)	30	(14.3)	·		
	Community hospital	27	(9.8)	24	(11.4)	3	(4.5)	
	Community practice	2	(0.7)	1	(0.5)	1	(1.5)	
	Private practice	101	(36.5)	92	(43.8)	9	(13.4)	
	Other	9	(3.3)	7	(3.3)	2	(3.0)	

* *Unpaired t-test, Fisher's exact, or χ^2^ test are used where appropriate*.

*NS, Not Significant*.

### Knowledge of SCID and Comfort in Meeting Patient Needs

As expected, participants in the non-SCID provider group reported a significantly lower mean rating of SCID knowledge than participants in the SCID provider group (x̄ = 4.8 vs. x̄ = 8.6, *p* < 0.0001). Respondents in the non-SCID provider group also indicated a significantly lower mean rating for comfort in meeting the needs of SCID patients than the respondents in the SCID provider group (x̄ = 4.4 vs. x̄ = 8.4, *p* < 0.0001). All responses were based on a 10-point scale.

### Information Needs

Nearly all respondents in the non-SCID provider group (93.9%) indicated that they were “very likely” to seek additional information about SCID if one of their patients were diagnosed. Most participants in the SCID-provider group (84.5%) sought additional information about SCID after having a patient with a diagnosis in their practice.

**Figure 1:**
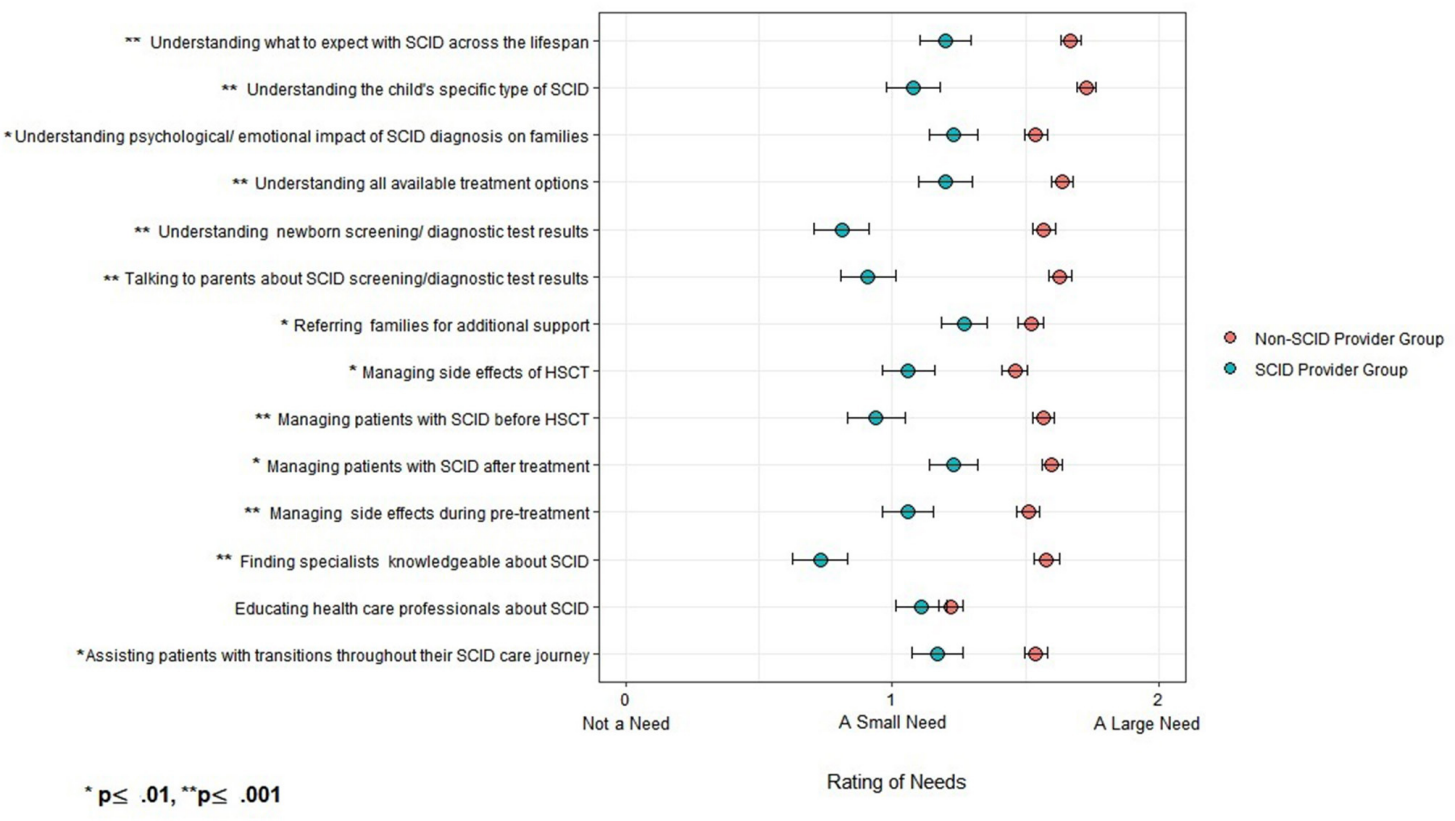
Ratings of informational needs in non-SCID provider group (*n* = 209) and SCID provider group (*N* = 67).

Providers were asked to rate whether informational needs presented in Figure 1 were 0 (“not a need”), 1 (“a small need”), or 2 (“a large need”). Overall, respondents in the non-SCID provider group had higher needs when compared with the respondents in the SCID provider group for all informational needs, except “educating other providers.” The largest needs identified by participants in the non-SCID provider group were “understanding specific type of SCID” (x̄ = 1.7) and “understanding what to expect across the lifespan” (x̄ = 1.7). Among participants in the SCID provider group, the highest rated informational need was “family support referrals” (x̄ = 1.3).

### Information Usefulness

Participants in the SCID provider group were asked to rate the quality of the SCID-related materials that they were familiar with (e.g., websites, research articles, and informational sheets). The quality of the materials was based on usefulness, trustworthiness, ease of understanding, and ease of locating. Usefulness was rated the highest (x̄ = 5.2) and ease of locating was rated the lowest (x̄ = 2.7) on a scale of 1 (“Extremely difficulty to locate”) to 7 (“Extremely easy to locate”).

### Preferred Information Sources

Table 2 describes preferred sources of SCID-related information by provider group. Overall, participants in the SCID provider group rated “other providers” and “peer-reviewed literature” as their preferred source of information (87.5% and 89.3%, respectively). The next likely source for information among this group was the Immune Deficiency Foundation (IDF), a patient advocacy organization (58.9%). About 70.7% in the non-SCID provider group reported that they would turn to the peer-reviewed literature for additional information. Around half of respondents in the non-SCID provider group said they would turn to professional organizations (56.5%), other providers (53.8%) or the clinical immunology listserv (50.0%) for additional information.

**Table 2:** Preferred information sources for non-SCID provider group (*N* = 184) and SCID provider group (*N* = 56).

	**Non-SCID provider group (*****N*** **= 184)**		**SCID provider group (*****N*** **= 56)**		**χ^2^ or Fisher's exact test**
	***N***	**%**	***N***	**%**	***p*** **-value**
Jeffrey Modell Foundation	8	4.4%	17	30.4%	<0.0001
SCID Angels for Life Foundation	17	9.2%	4	7.4%	NS
IDF^a^	53	28.8%	33	58.9%	<0.0001
NORD^b^	58	31.5%	11	19.6%	NS
ACMG ACT sheets^c^	38	20.7%	4	7.1%	<0.05
Other providers	99	53.8%	49	87.5%	<0.0001
Professional organizations	104	56.5%	27	48.2%	NS
Peer-reviewed literature	130	70.7%	50	89.3%	<0.01
Other	18	9.8%	2	3.6%	NS

a *Immune Deficiency Foundation*.

b *National Organization for Rare Disorders*.

c *American College of Medical Genetics and Genomics*.

*NS, Not Significant*.

### Preferred Information Formats

**Figure 2:**
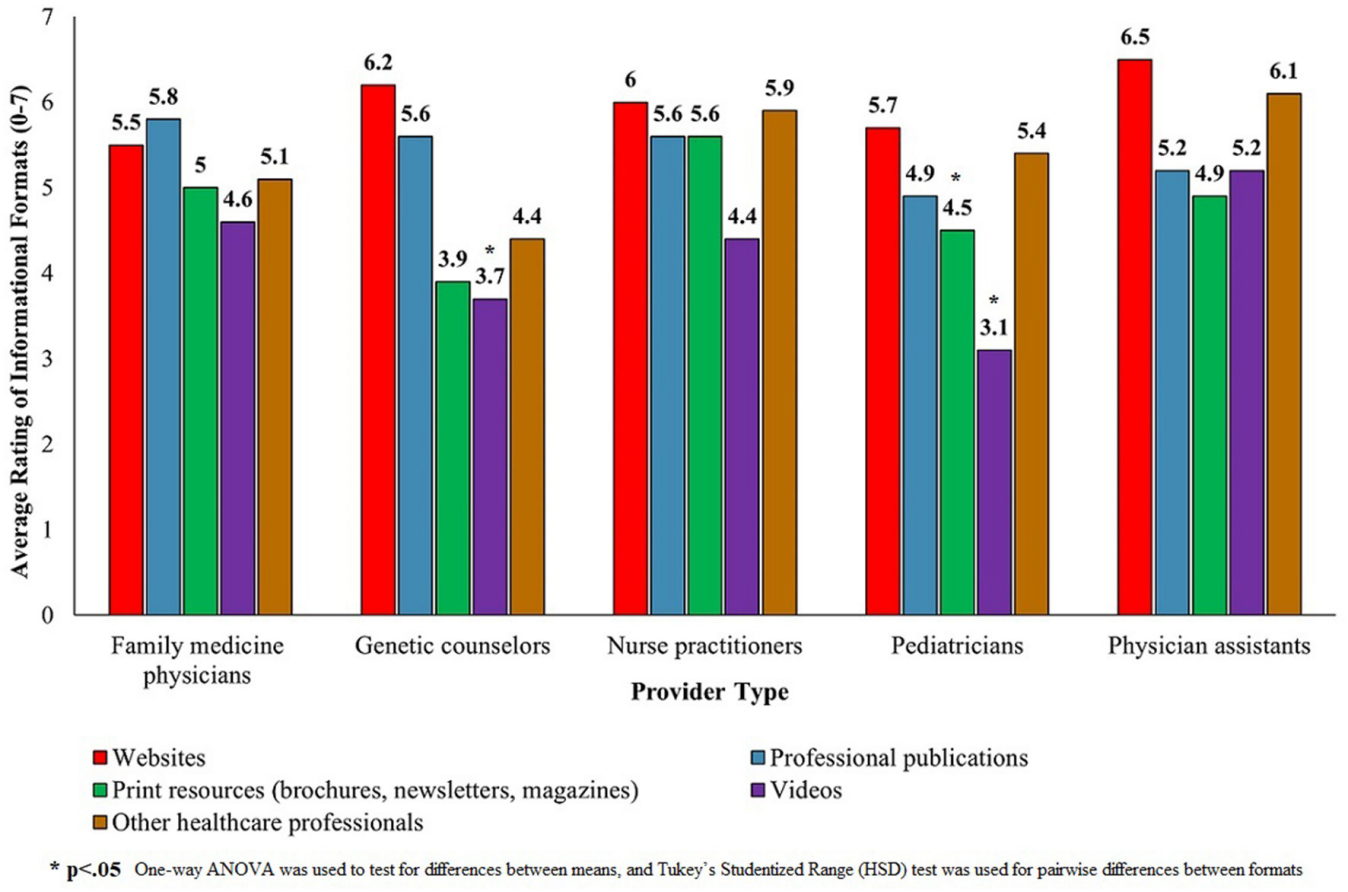
Ratings of informational formats among participants in the non-SCID provider group (*N* = 210).

When respondents in the non-SCID provider group were asked about their preferred format for SCID information, family medicine physicians rated reading a professional publication in a scientific journal the highest (x̄ = 5.8). Genetic counselors, nurse practitioners, pediatricians, and physician assistants preferred reading a website (Figure 2). For genetic counselors, watching a video was less preferred than using websites or professional publications (*p* < 0.05). For pediatricians, watching a video was less preferred to all other informational formats (*p* < 0.05), and print resources were less preferred than websites (*p* < 0.05). All responses were based on a 7-point scale.

## Discussion

Despite the implementation of nationwide newborn screening for SCID, there still exists a gap in knowledge about SCID treatment and management. Our study showed that providers who have treated none or only one patient with SCID (non-SCID provider group) reported having lower levels of SCID knowledge and higher levels of informational needs. These findings are likely correlated with the rarity of a SCID diagnosis. Pediatricians are often parents' first point of contact for SCID newborn screening results. In a study of follow-up care after a positive newborn screen for conditions such as phenylketonuria and congenital hypothyroidism, pediatricians and family physicians reported a lack of competence in discussing their child's condition with parents (14). Providers also reported uncertainties related to appropriate confirmatory testing and referral needs. It is important for pediatricians to have a strong knowledge of SCID to deliver clear communication to parents. Parents often report that succinct communication and smooth referral process to a specialist eased some of the uncertainties they experienced upon receiving a positive newborn screening result for SCID (1).

Respondents in the non-SCID provider group had a variety of informational needs, including understanding the different types of SCID and how SCID impacts the child's future. Other needs centered on interpreting screening results, making appropriate specialist referrals, and understanding SCID treatment options. Respondents in the SCID-provider group reported informational needs that focused on other areas of managing SCID, including referring parents to support services and overseeing patients' post-treatment. Our findings are consistent with providers' experiences in managing other genetic diseases (8, 15–18). Studies of physicians' experiences with cystic fibrosis, fragile X syndrome, and spinal muscular atrophy revealed gaps in knowledge related to these conditions and difficulties in providing parents with needed support (8, 18, 19).

Providers identified two preferred formats for receiving information about SCID—peer-reviewed publications and websites. There were variations in these preferences among provider types, with family medicine physicians preferring peer-viewed publications. These findings echo the conclusions of other provider-based assessments. A study of informational resource preferences among general pediatricians in the US identified academic literature as an important format of information (20). Another study of informational needs of general practitioners who manage patients with osteogenesis imperfecta, a rare inherited bone disease, identified websites as an important format of information (21). Our results have implications for the development and delivery of SCID-related resources to non-specialized practitioners who may treat SCID patients infrequently. Availability of such resources in accessible and user-friendly formats is an important step to helping physicians stay informed about rare diseases such as SCID. Given the providers' lack of knowledge and comfort around SCID management reported in our study and the lack of experience in providing follow-up care for rare diseases outlined in other studies, availability of easily accessible resources that provide point of care information may be key to improving providers' management of SCID (14). Currently, available websites such as the MedlinePlus Genetics of the U.S. National Library of Medicine contain information about causes, symptomology, inheritance, and treatment of more than 1,300 genetic conditions (22). The American College of Medical Genetics and Genomics provides ACT Sheets which contain information on various genetic conditions and are designed to assist clinicians in decision making (23). The ACT Sheets outline follow-up procedures after newborn screening, including methods of confirmatory testing, potential referrals, and a list of resources (14). However, previous studies have suggested that providers are often unaware that these resources are widely available (6). Given that providers in our study expressed concerns about locating SCID informational resources, there may be a need to increase education among medical providers on available informational resources for rare genetic diseases, including SCID. Availability of resources that are easily accessible, trustworthy, and user-friendly is vital to improving and maintaining providers' knowledge of SCID and increasing their comfort in treating patients with SCID.

### Study Limitations

Although this is a first study evaluating healthcare providers' SCID knowledge and informational needs, the findings presented here have limitations. First, our study participants represented a convenience sample which consisted of medical providers recruited through our partner organizations familiar with the SCID community and an online panel of healthcare providers. Therefore, our findings may not be generalizable to the entire medical community, both nationally and internationally. Additionally, our provider sample lacks racial and ethnic diversity. Therefore, our results may not reflect the informational needs and preferred resources formats of all providers. Third, we recruited a limited number of medical specialties, and therefore did not capture the knowledge and informational needs of all medical providers who may treat SCID patients. Finally, our study participants were asked to rate pre-specified categories of informational needs, sources, and formats. Thus, there may be additional unidentified categories SCID-related needs among providers. Notwithstanding these limitations, our study provides insight into SCID- related knowledge, current informational needs, and resource preferences among a range of medical specialties.

### Study Implications and Future Research

The results of our study provide important guidance for the development of informational resources for a variety of providers who treat patients with SCID. We have identified informational gaps among physicians related to interpreting SCID screening results, making specialist referrals, and understanding the various types of SCID and applicable treatments. Our study also identified preferred formats among providers for receiving SCID information such as websites and professional publications. These findings establish a foundation that will allow for the development of provider resources that address these informational gaps in preferred formats. The development and distribution of such resources may be used to increase both the providers' knowledge about SCID and their awareness of the availability of SCID-related resources. Further, our study may have implications for the development of similar informational resources for other rare genetic diseases. Future research may center on development and distribution of SCID materials which accommodate physicians' preference for resource formats and appropriately address SCID-related informational needs.

## Conclusion

Our study revealed knowledge gaps and varying informational needs around SCID treatment and management among medical providers. Study participants identified the need for reliable and trustworthy informational resources that address such topics as SCID treatment, causes, management, and referral practices. For providers who do not often encounter SCID patients, informational needs begin early in the SCID diagnostic journey. Providers identified preferred formats for receiving information about SCID, with some variation in preferences between provider types. Findings from this assessment will serve as a foundation for creating relevant, applicable, and easily accessible SCID-related informational resources for medical providers.

## Data Availability Statement

The raw data supporting the conclusions of this article will be made available by the authors, without undue reservation.

## Ethics Statement

The studies involving human participants were reviewed and approved by RTI International. Written informed consent for participation was not required for this study in accordance with the national legislation and the institutional requirements.

## Author Contributions

OK was responsible for conducting the analysis and writing and reviewing the manuscript. AC, AG, and MR were involved in survey construction, data conceptualization and analysis, and writing and reviewing the manuscript. MR conceptualized and designed the study. All authors approved the final manuscript and agreed to be accountable for all aspects of the work.

## Funding

This project was supported by the Health Resources and Services Administration (HRSA) of the U.S. Department of Health and Human Services (HHS) as part of an award totaling $2.97 million with 0% financed with non-governmental sources.

## Author Disclaimer

The contents are those of the author(s) and do not necessarily represent the official views of, nor an endorsement, by HRSA, HHS, or the U.S. Government.

## Conflict of Interest

AC was employed by Immune Deficiency Foundation.

The remaining authors declare that the research was conducted in the absence of any commercial or financial relationships that could be construed as a potential conflict of interest.

## Publisher's Note

All claims expressed in this article are solely those of the authors and do not necessarily represent those of their affiliated organizations, or those of the publisher, the editors and the reviewers. Any product that may be evaluated in this article, or claim that may be made by its manufacturer, is not guaranteed or endorsed by the publisher.
