# Supplementary material for: Severe Combined Immunodeficiency: Knowledge and Information Needs Among Healthcare Providers
Source: Front Pediatr. 2022 Feb 21;10:804709. doi: 10.3389/fped.2022.804709 (PMC8899266; doi:10.3389/fped.2022.804709)
Supplement: Supplementary file 1 [file Data_Sheet_1.docx]

# SCID Provider Survey

### **Section 1: Demographics**

1. What is your sex?
   1. Male
   2. Female
   3. Other
   4. Prefer not to say
2. What is your race? [check all that apply]
   1. American Indian or Alaskan Native
   2. Asian
   3. Black or African American
   4. Native Hawaiian or Other Pacific Islander
   5. White
   6. Prefer not to say
3. What is your ethnicity?
   1. Hispanic or Latino
   2. Not Hispanic or Latino
   3. Prefer not to say
4. What is your age?
   1. _____[0-99] TERMINATE IMMEDIATELY IF s4=0, otherwise terminate at the end of screener for s4<18
5. Please indicate your primary role or specialty area [PN: TERMINATE IMMEDIATELY IF F-J]
   1. Pediatrician
   2. Family medicine physician
   3. Genetic counselor
   4. Nurse Practitioner
   5. Physician assistant
   6. Allergist-immunologist
   7. Rheumatologist
   8. Oncologist-hematologist
   9. Bone marrow transplant physician
   10. Other (please specify)
6. How many years of experience do you have in your primary role or specialty area?
   1. _____ [0-99] TERMINATE IMMEDIATELY OF s6=0, OTHERWISE TERMINATE AT THE END OF SCREENER IF <3 or >30
7. In which type of practice setting do you spend most of your work time?
   1. Private practice
   2. Community clinic
   3. Community hospital
   4. Academic hospital
   5. Other (please specify)

10. Please tell us the state where you spend most of your work time? [states drop down list]

1. Please tell us the zip code for the practice where you spend most of your work time?
   1. **5-digit field**
2. Have you ever had a patient with a diagnosis of severe combined immunodeficiency (SCID) or a positive newborn screen for SCID? SCID is a potentially fatal primary immunodeficiency in which there is combined absence of T-lymphocyte and B-lymphocyte function.
   1. No **Go to Section 2**
   2. Yes **Go to Section 3**

### **Section 2: Questions for providers who do not have a patient with SCID**

Please read the following hypothetical case study and imagine this was one of your patients. Please use this example to help you respond to the subsequent questions.

Patient A is a male infant who had a positive newborn screen for SCID in a US state which routinely performs newborn screening. He was born at 39 weeks gestational age via spontaneous vaginal delivery without prenatal or delivery complications. Mother initiated breastfeeding shortly after birth. Within 24 hours of birth, the infant was examined by the on-call pediatrician from the office that his 3-year old older sister goes to. The newborn assessment was normal, and the infant looked completely healthy. He was discharged home on day of life 2. His newborn screen for SCID with T cell receptor excision circles (TRECs) indicated 56 cells/μL, suggesting a SCID diagnosis. His pediatrician called the family on day of life 5 to inform them of the newborn screen result. The infant was subsequently referred for further immunological evaluation on day of life 8. Upon retesting, absolute lymphocyte counts were low, including near absence of T cells and low NK and B cell counts. Notably, the ratio of CD4+CD45RA+ naïve to total CD4+ T cells was also very low (11%). These findings suggested abnormal thymic T cell maturation.

1. How knowledgeable are you about SCID?
   1. Not at all knowledgeable [**slider option: scale of 1-10**] Very knowledgeable
2. How comfortable are you in meeting the needs of patients with SCID, overall?
3. Not at all comfortable [**slider option: scale of 1-10**] Very comfortable
4. How likely would you be to seek additional information about SCID if one of your patients was diagnosed?
   1. Not at all likely **Go to question #4**
   2. Somewhat likely **Go to question #5**
   3. Very likely **Go to question #5**
5. Why would you not seek additional information about SCID? Please select all that apply.
   1. I would refer the patient to a specialist for their care **Go to question #6**
   2. I would refer the patient to another primary care provider **Go to question #6**
   3. I already know a lot about SCID **Go to question #6**
   4. I do not have time to seek additional information **Go to question #6**
   5. Other (please specify) **Go to question #6**
6. Where would you seek additional information if you had a patient diagnosed with SCID? Please select all that apply.
   1. Other providers
   2. Peer-reviewed literature
   3. Professional organizations, such as the American Academy of Allergy, Asthma, and Immunology (AAAAI) or the American Academy of Pediatrics (AAP)
   4. American College of Medical Genetics ACT sheet
   5. National Organization for Rare Diseases (NORD)
   6. Immune Deficiency Foundation
   7. SCID Angels for Life Foundation
   8. Jeffrey Modell Foundation
   9. I don’t know
   10. Other (please specify)
7. How likely would you be to seek additional information about SCID using a search engine?
   1. Not at all likely **Go to question #8**
   2. Somewhat likely **Go to question #7**
   3. Very likely **Go to question #7**
8. What search terms would you use when looking for information on SCID?
   1. **Open text box**

8. If a new patient with SCID came into your practice today, please consider whether any of the following would be not a need, a small need, or a large need for you.

|  | Not a need | A small need | A large need |
| --- | --- | --- | --- |
| Understanding the child’s newborn screening and/or diagnostic test results |  |  |  |
| Understanding the child’s specific type of SCID |  |  |  |
| Talking to parents about the results of the SCID screening or diagnostic test |  |  |  |
| Explaining to parents what to expect with SCID across the lifespan |  |  |  |
| Explaining all available treatment options, including the risks/benefits of treatment and chances of treatment success to parents |  |  |  |
| Referring patients to specialists who are knowledgeable about SCID and SCID treatments |  |  |  |
| Managing your patient’s health care before they receive treatment |  |  |  |
| Managing any side-effects of pretreatment or conditioning |  |  |  |
| Managing side effects of hematopoietic stem cell transplantation or other treatments |  |  |  |
| Managing your patient’s health care needs after treatment |  |  |  |
| Helping families manage the psychological or emotional impact of a SCID diagnosis |  |  |  |
| Referring families for additional support |  |  |  |
| Educating other health care professionals about SCID |  |  |  |
| Assisting patients throughout all phases of their SCID health care journey |  |  |  |

9. What types of materials would you want if a child in your practice was diagnosed with SCID?

[**Open-ended response**]

10. Please indicate how interested you would be in learning about SCID through each of the following methods: (slider from 0 = not interested to 7 = extremely interested)

- 1. Reading a website
  2. Reading a professional publication in a scientific journal
  3. Reading other printed information (for example: brochure, newsletter, magazine, or other publication)
  4. Watching a video
  5. Talking with other healthcare professionals
  6. Other (please specify)

11. In your opinion, who should be responsible for providing information to patients and their families? Please select all that apply.

1. Pediatrician or other primary care provider
2. SCID specialist, such as an Allergist-immunologist or clinical immunologist
3. Patient advocacy organizations
4. Other (please specify)

**🡪Go to Section 4**

###

### **Section 3: Questions for providers who have a patient with SCID (and respondent type is pediatrician, family medicine, genetic counselor, nurse practitioner, or physician assistant)**

1. How many patients with SCID have you treated?
   1. ____ [1-999]
2. How recently have you provided care for a patient with SCID?
   1. In the last year
   2. Between 1 and less than 3 years ago
   3. Between 3 and less than 5 years ago
   4. More than 5 years ago
3. How knowledgeable are you about SCID?
   1. Not at all knowledgeable [**slider option: scale of 1-10**] Very knowledgeable
4. How comfortable are you in meeting the needs of patients with SCID?
   1. Not at all comfortable [**slider option: scale of 1-10**] Very comfortable
5. Have you sought additional information about SCID since having a patient with a diagnosis in your practice?
   1. No **Go to question #7**
   2. Yes **Go to question #6**
6. Where have you sought additional information about SCID? Please select all that apply.
   1. Other providers, such as SCID specialists
   2. Peer-reviewed literature
   3. SCID-specific professional organizations, such as the American Academy of Allergy, Asthma, and Immunology (AAAAI)
   4. Clinical Immunology Society listserv
   5. Other professional organizations, such as the American academy of Pediatrics
   6. American College of Medical Genetics ACT sheets
   7. National Organization for Rare Diseases (NORD)
   8. Immune Deficiency Foundation
   9. SCID Angels for Life
   10. Jeffrey Modell Foundation
   11. I don’t know
   12. Other (please specify)
7. Have you ever sought additional information about SCID using a search engine?
   1. No **Go to question #9**
   2. Yes **Go to question #8**
   3. I don’t remember **Go to question #9**
8. What search terms did you use when looking for information on SCID?
   1. **Open text box**
9. If a new patient with SCID came into your practice today, please consider whether any of the following would be a large need, a small need, or not a need for you.

|  | Not a need | A small need | A large need |
| --- | --- | --- | --- |
| Understanding the child’s newborn screening and/or diagnostic test results |  |  |  |
| Understanding the child’s specific type of SCID |  |  |  |
| Talking to parents about the results of the SCID screening or diagnostic test |  |  |  |
| Explaining to parents what to expect with SCID across the lifespan |  |  |  |
| Explaining all available treatment options, including the risks/benefits of treatment and chances of treatment success to parents |  |  |  |
| Referring patients to specialists who are knowledgeable about SCID and SCID treatments |  |  |  |
| Managing your patient’s health care before they receive treatment |  |  |  |
| Managing any side-effects of pretreatment or conditioning |  |  |  |
| Managing side effects of hematopoietic stem cell transplantation or other treatments |  |  |  |
| Managing your patient’s health care needs after treatment |  |  |  |
| Helping families manage the psychological or emotional impact of a SCID diagnosis |  |  |  |
| Referring families for additional support |  |  |  |
| Educating other health care professionals about SCID |  |  |  |
| Assisting patients throughout all phases of their SCID health care journey |  |  |  |

1. Have your patients with SCID or their caregivers asked you for information about SCID?
   1. No **Go to question #12**
   2. Yes **Go to question #11**
2. What type of information have they asked for?
   1. **Open-ended response**

12. Have you ever given caregivers information on SCID?

- 1. Yes **Go to question #13**
  2. No **Go to question #14**

13. Please rate your experiences with SCID-related materials that you have given to families on the following dimensions:

- 1. Usefulness (**1 = Not at all useful to 7 = Extremely useful)**
  2. Ease of understanding **(1 = Extremely difficult to 7 = Extremely easy)**
  3. Difficulty in locating **(1 = Extremely difficult to 7 = Extremely easy)**
  4. Trustworthiness **(1 = Very untrustworthy to 7 Very trustworthy)**

**14. Do you have any additional comments to share?**

**Open-ended response**

Thanks for your time in completing the survey.
